# Synthetic-Polymer-Based Cardiac Patches for MI-Induced Heart Failure Treatment: A Review

**DOI:** 10.3390/biom16040580

**Published:** 2026-04-14

**Authors:** Ahmed Eliwa, Mohamed K. Abbas, Maryam Al-Ejji, Khadija Zadeh, Hamda Aboujassoum

**Affiliations:** 1 College of Medicine, Qatar University, Doha P.O. Box 2713, Qatar; 2 Internal Medicine Residency Program (IMRP), Division of Internal Medicine, Hamad Medical Corporation, Doha P.O. Box 3050, Qatar; 3 Center for Advanced Materials (CAM), Qatar University, Doha P.O. Box 2713, Qatar; maryam.alejji@qu.edu.qa (M.A.-E.); khadija_zadeh@qu.edu.qa (K.Z.); 4 Laboratory Animal Research Center (LARC), Qatar University, Doha P.O. Box 2713, Qatar; hbojassom@qu.edu.qa

**Keywords:** cardiac patches, polymers, biomaterials, biocompatibility, synthetic polymers

## Abstract

Myocardial infarction (MI) is one of the prevalent cardiovascular diseases, which is caused by obstruction of one or more coronary arteries, leading to cardiac tissue ischemia and death. One of the main consequences of MI is heart failure, which is defined as dysfunction of the heart muscle to pump blood into peripheral organs. Cardiac patches have drawn a lot of interest as a potentially effective way to restore damaged cardiac tissue and enhance its functionality. They are polymer-based scaffolds designed to be implanted on the heart surface, and they have shown a significant therapeutic effect in the treatment of MI by improving cardiac function and providing mechanical support for the infarction site by the delivery of various bioactive substances or cells. Several biomaterials with specific mechanical and chemical characteristics have been widely used as a scaffold in the process of fabricating cardiac patches. In this study, we focus on the latest developments in the manufacturing of synthetic-polymer-based cardiac patches used to treat heart failure induced by myocardial infarction. We describe the mechanical and chemical characteristics of several synthetic polymers and highlight the main benefits and drawbacks of each type. An overview of the major challenges and the future development directions in the field of cardiac patches is also highlighted.

## 1. Introduction

Heart failure (HF) is a prevalent clinical syndrome characterized by symptoms arising from a structural or functional cardiac abnormality that hinders the ventricle’s capacity to fill or expel blood. It is identified by the presence of current or prior characteristic symptoms, such as breathlessness (dyspnea) at normal or low-level exertion, fatigue, and fluid retention [1]. HF is considered a global epidemic, with an estimated 64.3 million cases worldwide in 2017 [2]. The impact of HF on global health care costs is worrisome. In 2012, the overall annual economic burden of heart failure in the United States was estimated at $30.7 billion, encompassing both healthcare expenditures and productivity losses. Projections indicate that by 2030, expenses will have increased by 127% to $69.8 billion, or about $244 for every adult in the country [3].

HF is a multifaceted and intricate illness that may have multiple underlying causes. The etiology of heart failure differs considerably between regions worldwide. Considerably, it can arise from a variety of underlying pathological processes [4]. Myocardial infarction (MI) is a significant risk factor for HF. Cardiovascular disease (CVD), including MI, was the primary cause of 8.9 million deaths in women and 9.6 million deaths in men in 2019, accounting for over one-third of all fatalities worldwide. Individuals under 65 years of age accounted for approximately 6.1 million deaths, emphasizing the considerable impact of cardiovascular disease on the younger and middle-aged population [5]. MI refers to the death of cardiac myocytes due to ischemia, which arises from an imbalance between supply and demand of blood perfusion [6]. Modifiable risk factors for MI include hypercholesterolemia, diabetes, hypertension, obesity, and smoking [7]. Despite the significant advancements in the management of acute MI and coronary artery disease over the last 20 years, MI remains to be the leading cause of HF [8]. The pathogenesis in which MI can potentially cause HF is complex and involves several mechanisms, including myocardial compromise brought on by myocardial necrosis, myocardial stunning, and mechanical dysfunction, such as rupture of the papillary muscles, ventricular septal defect, and rupture of the ventricular free wall [9]. The primary intervention for MI is coronary reperfusion, which acts to restore blood perfusion to dead myocardial cells. However, the process of reperfusion itself causes further damage to the myocardium through the generation of reactive oxygen species (ROS) [10]. The development of HF in the setting of MI is also influenced by the inflammatory response to myocyte damage. All these factors consequently cause structural damage to the heart and lead to weakened myocardial fibers that are not able to pump blood effectively.

The New York Heart Association (NYHA) has classified HF patients into four classes depending on the severity of symptoms caused by the failing heart. Each class has specific management criteria [11]. Currently, the mainstay of HF management is improving the symptoms and preventing further deterioration [12]. This can be achieved by using pharmacological agents like diuretics, beta blockers, and renin–angiotensin system inhibitors, along with lifestyle modifications, such as cessation of smoking and abstinence from alcohol consumption [13]. However, these medications are effective in improving quality of life for mild and moderate HF stages only. In addition, they work on reducing the severity of symptoms but they do not have a significant impact on the trajectory of the disease [11]. In the advanced HF stage, patients become refractory to conventional HF medications despite optimum pharmacotherapy and may require other modalities. However, ventricular assist devices (VADs) or heart transplantation are the only two alternatives available for treating end-stage HF [14]. While there is a wide range of VADs, the majority of them necessitate direct contact with the patient’s blood; as a result, thromboembolic events, the requirement for anticoagulation, immunological reactions, and hemolysis are constant concerns [15].

Although heart transplantation has been the standard method of treatment for end-stage HF patients, there are still serious challenges which limit its application and success [16]. One of these drawbacks is the scarcity of donor organs, which significantly reduces the number of patients eligible for heart transplants [17]. Cardiac allograft vasculopathy (CAV) and cancer are serious complications, which compromise the recipients long-term survival after the procedure [18]. Malignancies (including solid tumors and lymphomas) are more common in heart transplant recipients than in kidney transplant recipients [19]. This is likely because heart transplant recipients require more intensive immunosuppression overall, which increases the likelihood of malignancy [19]. Drug-induced complications from chronic immunosuppression are another major challenge after heart transplantation treatment, which increase the risk of morbidity and mortality in patients receiving heart transplants [20]. Collectively, these challenges explain why, despite a nearly 20% rise in the number of new adults on the waiting list, there has not been a rise in the number of adult heart transplants performed over the past ten years (about 4000 documented transplants worldwide per year) [21]. Therefore, it has become crucial to develop an efficient therapeutic modality that aids in repairing the injured myocardium and preventing the progression to end-stage heart failure.

Researchers have investigated the application of a transient, local patch to the infarcted ventricle’s surface, formally named a cardiac patch [22]. This patch has shown an evident superiority in helping the damaged myocardium regain functionality [23]. In this review, we highlight the most recent advancements in the development of cardiac patches composed of synthetic polymers that are utilized to treat myocardial-infarction-induced HF. We discuss the advantages and disadvantages and biomechanical characteristics of the main synthetic polymers used for manufacturing cardiac patches, which are: poly(glycerol-sebacate) (PGS), polyurethane (PU), polycaprolactone (PCL) and poly(L-lactic acid) (PLLA). Additionally, the cellular mechanisms of promoting cardiac recovery of various cardiac patches are also explained. Lastly, we provide a brief outlook on the challenges that this field may face in the future and potential remedies.

## 2. Cardiac Patch: Functions, Composition and Characteristics

**Figure 1:**
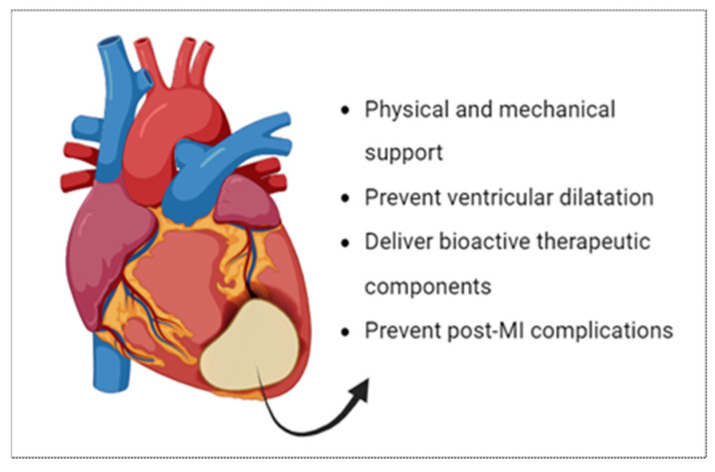
The functions of a cardiac patch implanted on an infarcted heart tissue.

As previously mentioned, cardiac patches are polymeric porous scaffolds constructed to serve as a bandage implanted on the infarcted failing heart. The benefit of these patches is accomplished through various methods. The main function of these scaffolds is to provide adequate physical and mechanical support to the infarcted heart [24]. This could be achieved by fabricating a scaffold that mimics the mechanical dynamics of a human heart and restores its effective contractile function. After myocardial damage or infarction, ventricular remodeling takes place, which is a complex process characterized by cavity dilation because of the eccentric hypertrophy of cardiac fibers [25]. The necrotic region becomes more prone to distension due to the loss of structural support and, consequently, more susceptible to ventricular dysfunction and failure [26]. Therefore, one of the important functions of cardiac patches is to act as barrier to limit ventricular dilatation and post-myocardial infarction expansion [22]. In addition, cardiac patches have a vital biological function, in which they serve as a carrier to deliver various bioactive therapeutic components, including induced pluripotent stem cells, human cardiac progenitor cells, bioactive growth factors, and nanozymes [27]. Consequently, they induce myocardium healing and repair through preserving the optimum microenvironment for the cells to undergo differentiation and proliferation. Collectively, these multiple functions act to promote ventricular contractile capacity and improve overall heart function. Figure 1 summarizes the main functions of cardiac patches.

In addition to the previously mentioned benefits, cardiac patches offer several features compared to other modalities of heart failure treatment. One feature is the patient-specific design of the scaffold, enabling customized cell placement to achieve targeted therapeutic outcomes [28]. Another feature is that they offer complete surface coverage to the damaged heart tissue, which is required for applications involving mechanical support and drug delivery. Lastly, they protect the heart from post-myocardial infarction complications, including ventricular wall rupture, ventricular aneurysm formation, and ventricular arrythmias [29].

To fabricate an ideal cardiac patch, there are several crucial parameters that should be taken into consideration. The fundamental parameters of a fully integrated cardiac patch are materials that can sustain biological activity, prevent a negative host immune response, and endure the dynamic forces of the heart, such as contraction, torsion, tension, and shear stresses [30]. However, the primary necessity for an optimal patch is to mimic the characteristics of the native heart tissue, which include strong resilience, excellent cardiomyocyte attachment, and synchronous contraction [31]. Therefore, the biomaterials used to fabricate cardiac patches should have topographical, mechanical, and biochemical characteristics that are comparable to those of the native heart tissue. The first important parameter is biocompatibility. According to Ratner, biocompatibility is the ability of a material to cause a particular organism reaction [32]. These adverse reactions elicited against a foreign implant could be immunological, toxicological, thrombotic, or hemolytic. In order to prevent adverse foreign body reactions when the resulting cardiac patches are implanted on the heart surface, the materials used in their manufacture must possess a significant level of biocompatibility [33].

Having appropriate mechanical properties is another essential requirement when fabricating cardiac patches. One of the most important mechanical properties is possessing high compliance to adapt to the myocardium’s long-term elasticity in dynamic circumstances [23]. Given that the heart performs contraction during the systolic phase and relaxation during the diastolic phase, the biomaterial should withstand the mechanical demands for the ventricle, where the stiffness is 10–20 kPa and 200–500 kPa at the beginning and end of diastole respectively [34]. Another vital mechanical characteristic is having adequate porosity. To increase the therapeutic efficacy of cardiac patches, the biomaterial scaffolds should be porous in order to preserve and release cells or bioactive components onto the damaged myocardial area (a patch filled with cells typically has a porosity of more than 90% and pore sizes greater than 50 μm) [35]. Furthermore, the mechanical microstructure of cardiac patch should have an ideal configuration that promotes the alignment and elongation of cardiomyocytes, which take place during the process of repair after implantation [36]. In addition, it is crucial to have suitable surface chemistry to enhance cell adhesion and growth as well as the capacity for synchronous contraction to enhance bioelectric signal conductivity and prevent severe arrhythmias in vivo [37].

**Figure 2:**
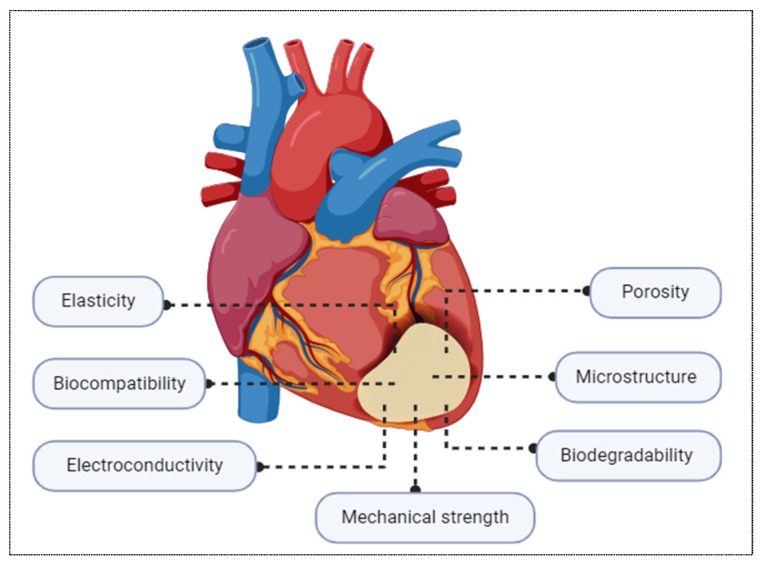
Demonstration of a cardiac patch implanted on an area of myocardial infarction and the main requirements for a successful patch.

Biodegradability is one of the fundamental parameters which should be assessed when fabricating cardiac patches [23]. After the successful regeneration process of myocardial cells, the long-term existence of the patch can cause fibrous capsule formation and chronic inflammatory response in the cardiac tissue [38]. Therefore, to prevent detrimental effects in the body, the biomaterials must be biodegradable, and their rate of degradation must be in line with the formation of new tissue. Additionally, the degradation products must be biocompatible and have no negative side effects [39]. In addition to being both biocompatible and biodegradable, the patch’s mechanical characteristics, porosity, and microstructure should all adequately mimic the physiological norms of the heart in order to perform as a functional therapeutic component. Consequently, the patch can host a range of cells and biological elements that reside in the tissue for a successful repair and healing process. Figure 2 illustrates the main requirements for cardiac patch fabrication.

## 3. Synthetic-Based Polymers

Synthetic polymeric scaffolds stand out as top contenders for cardiac patch tissue engineering owing to their customizable nature and ease of fabrication processes, enabling tailored designs to precisely mimic native cardiac tissue requirements [40]. Polymers offer a wide array of mechanical properties and exhibit excellent biocompatibility, with degradation rates that are easily modifiable [41]. Additionally, synthetic polymers boast resilience, porosity, and microstructural intricacies that can be fine-tuned to closely resemble natural cardiac tissues [42]. Despite potential challenges like diminished cell adhesion and scaffold integration inherent in polymer biomaterials, their widespread acceptance persists due to feasible modifications such as the incorporation of stimuli and growth factors [43]. Polymer chain variability is rooted in factors such as chemical structures, molecular weights, molecular weight distribution, and attachable functional groups [44]. The design of cardiac scaffolds demands a delicate balance of elasticity and mechanical strength to withstand the dynamic cardiac milieu. Many advanced cardiac scaffolds integrate blends of polymers to achieve these critical properties, effectively amalgamating the mechanical characteristics of diverse polymers and yielding scaffolds with a plethora of desirable attributes. The fabrication strategies employed for these material patches exhibit significant diversity, influenced by a blend of material properties and physiological requisites. These materials can undergo various processing methods to produce scaffolds with distinct morphologies. Techniques such as phase separation and salt leaching can generate porous scaffolds [45]. Electrospinning stands out as a prevalent method for crafting nano- or sub-micro-fibrous scaffolds [46], although nano-fibrous morphology can also be achieved through phase separation. Leveraging reproducible synthesis methods, synthetic materials with consistent physical and mechanical properties demonstrate promise in meeting clinical standards [47]. Numerous synthetic materials for tissue engineering have been extensively investigated. The main synthetic polymers that will be covered under this section are: polycaprolactone (PCL), poly-(L-lactic) acid (PLLA), and polyurethanes (PU), and poly(glycerol-sebacate) (PGS).

### 3.1. Poly(ε-Caprolactone)-Based Patches (PCL)

**Figure 3:**
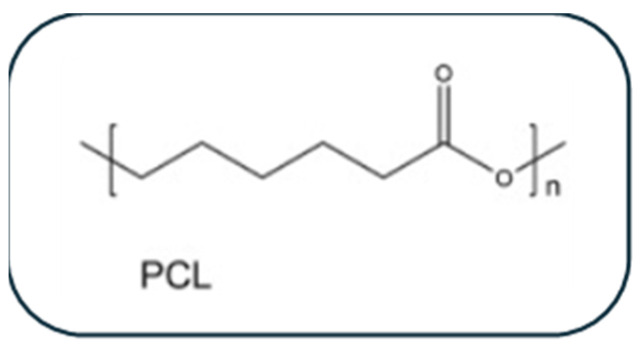
Chemical structure of PCL.

Polycaprolactone (PCL) is perceived as a class of biodegradable aliphatic polyesters. Owing to the fact that it has a low melting temperature and remarkable blend compatibility, this semi-crystalline polymer has high solubility at room temperature and could be easily processed [48]. As one of the first commercially accessible synthetic polymers, PCL has a wide range of mechanical and biodegradation characteristics that may be precisely controlled by adjusting the local environmental driving forces (enzymes, microbes, hydrolysis, etc.) [49]. The molecular weight, polydispersity index, and degree of crystallinity all impact the physical, mechanical, and thermal properties and biodegradation duration. Therefore, many of the practical biomedical applications depend on controlling these variables during PCL synthesis [48]. PCL stands out for its superior structural support, affordability, and ease of availability [50]. Consequently, It has been extensively used as a synthetic biomaterial in numerous biomedical applications due to its remarkable biocompatibility and biodegradability [51]. In addition, it can be combined with other substances, copolymers, and composites to fabricate cardiac patches that have essential mechanical and physicochemical characteristics. The chemical structure of PCL is demonstrated in Figure 3.

Biodegradability is one of the important characteristics of PCL when being implanted into the human body. It is theoretically possible for a PCL patch to completely integrate into the myocardium after being implanted on the heart without leaving behind any foreign synthetic material [52]. Over time, the gradual breakdown of PCL would provide mechanical support for tissue integration, enabling the development of a fully developed, contractile tissue. According to the literature, PCL’s degradation time is about 2 to 4 years [53]. In contrast to other polyesters, PCL degrades more slowly due to the hydrophobic nature of its CH2 moieties in its repeating unit [52]. Therefore, the delayed degradation time of PCL scaffolds provides sufficient mechanical strength for the healing process of the damaged cardiac cells to take place. The mechanism of PCL’s degradation at blood pH is principally achieved by hydrolysis of ester groups, resulting in chain scission at random locations [54]. As a result of this process, the molecular weight is gradually reduced, and fragmentation of the polymer chain takes place. Subsequently, the fragments may undergo metabolism via the tricarboxylic acid cycle or be eliminated through urination [55]. Therefore, it is generally accepted that PCL and its breakdown products are safe for implantation and have no discernible effect on in vivo immune responses.

**Figure 4:**
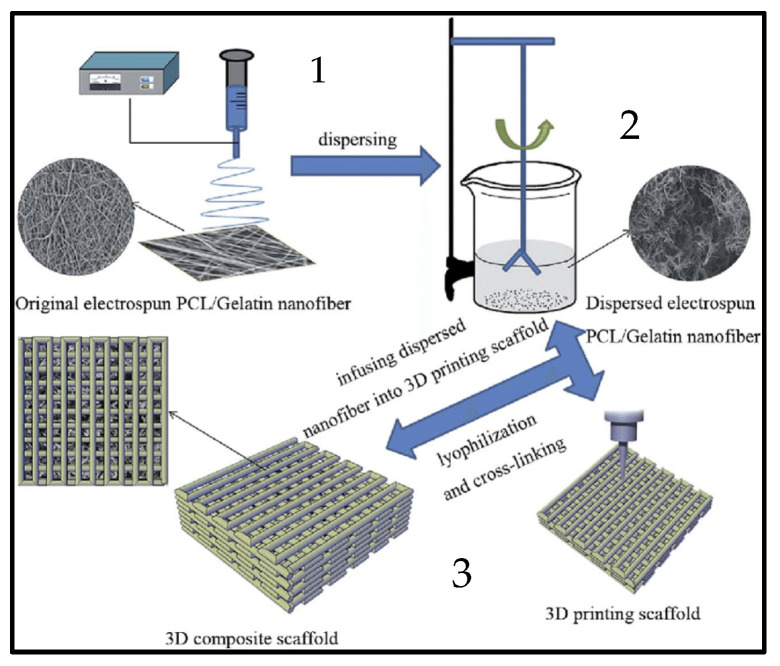
Schematic of the composite scaffold with electrospinning and 3D printing technology. Step (1): PCL/gelatin was fabricated with nanofibers and then treated to disperse the nanofibers; step (2): PCL was printed on the 3D scaffold; step (3): the dispersed nanofibers were filled into the meshes of the 3D-printed scaffold to fabricate the 3D composite scaffold.

Another essential feature of PCL is biocompatibility and low immunogenicity. Because PCL is considered to be nonimmunogenic, there is little-to-no inflammatory response following PCL implantation [56]. The immune response against PCL implantation in vivo has been thoroughly assessed in the literature. Following PCL-PLLA blend scaffold implantation into rabbit femurs, Sadiasa et al. observed no inflammatory response, as determined by histological examination, which also showed no adherent macrophages and good tissue growth surrounding the implant [57]. Another study done by Veiseh et al. [58] aimed to compare the immunological response against small and large PCL implants in vivo. Remarkably, they discovered that larger PCL implants showed reduced fibrosis and less recruitment of neutrophils and macrophages. In another research experiment done by Gil-Castell et al. [59] the performance of nanofibrous electrospun scaffolds composed of PCL and gelatin (Ge) was assessed both in vitro and in vivo. Figure 4 demonstrates a schematic diagram of the electrospinning technique used to fabricate PCL scaffolds [60]. Their results demonstrated when PCL/Ge 40/60 scaffolds were implanted, the infarcted myocardium’s scar tissue was decreased, the left ventricle wall thickness was significantly reduced, and the scaffolds were fully assimilated in 15 days. In addition, they assessed the biocompatibility of PCL scaffolds during in vivo implantation. Using a pyrogen test, the levels of IL-1β, IL-6, IL-10, and TNF gene expression were measured following contact with the scaffolds in order to evaluate the acute inflammatory response. After 15 days of implantation, none of the scaffolds showed signs of chronic inflammatory processes [59]. This demonstrates that when implanted in vivo, PCL scaffolds have a regulated rate of degradation and are extremely biocompatible [61]. When considered collectively, the outcomes demonstrate that using these scaffolds for fabricating cardiac patches is a reasonable strategy to reduce myocardial damage following an acute myocardial infarction [62].

Furthermore, PCL has shown a significant role in the process of repairing and healing infarcted cardiac tissue through different cellular mechanisms. Restoring blood circulation to the infarcted myocardium, as previously mentioned, results in the production of reactive oxygen species (ROS), which induces oxidative stress and further cardiac damage [63]. Research has demonstrated that PCL scaffolds combined with cerium oxide nanoparticles can effectively scavenge cellular ROS, protecting cardiac cells from oxidative injury [64]. Jain et al. [65] utilized electrospinning to fabricate a nanostructured polymer scaffold from PCL nanofibers coated with cerium oxide. When primary cardiomyocytes were exposed to H_2_O_2_-induced oxidative stress, ROS levels significantly decreased in the cells grown on cerium-oxide-decorated PCL nanofibers. Ultimately, the results demonstrated the potential of PCL nanofibers coated with cerium oxide as an antioxidant and anti-hypertrophic cardiac patch.

In addition, several mechanisms have been established on how PCL scaffolds promote cardiac tissue function post-implantation. One of the most important mechanisms is promoting adhesion and attachment of cardiac cells to PCL-based scaffolds through chemical modification. It has been proven that there is a direct relationship between the degree of hydrophilicity of PCL and the strength of cardiac cell adhesion to the scaffold [38]. Spearman et al. [66] used PCL films treated with sodium hydroxide (NaOH) to encourage alkaline-mediated hydrolysis, which consequently increased the hydrophilicity of the PCL. Pretreatment with NaOH resulted in a considerable increase in adherent CMs per unit area (1568 ± 126 cells/mm^2^, 2880 ± 439 cells/mm^2^, and 3623 ± 456 cells/mm^2^ for PCL with 0, 24, and 48 h of NaOH pretreatment, respectively). Moreover, oxygen plasma treatment is another process that has been utilized to improve cardiomyocyte adhesion to PCL. In this process, hydrophilicity is achieved by transporting oxygen-containing groups to the surface of the polymer [67]. Another mechanism by which PCL scaffolds can improve cardiac tissue functionality is through supporting cardiac contractility and mimicking the mechanical stiffness of the native human heart (∼10 kPa) [68]. Modulation of PCL mechanical stiffness can be achieved through various biofabrication techniques. Yeong et al. [69] created a porous PCL scaffold through the use of a selective laser sintering approach, which effectively reduced the value of Young’s modulus of the PCL scaffold. The PCL scaffold’s stiffness was considerably reduced in comparison to bulk PCL, since its cross-sectional area was expanded without any additional mass being added. Yeong et al. reported a tensile stiffness of 0.43 ± 0.15 MPa and a compressive stiffness of 342 kPa for 85% porosity. By achieving a mechanical stiffness similar to the human heart, the patch fabricated from PCL can regain the functional contractility of the weakened myocardium, which will help in pumping blood more efficiently.

One essential element in which PCL scaffolds support the electromechanical function of cardiac cells is via anisotropy [70]. Anisotropy is defined as the characteristic of directional dependence. The direction of the myocytes determines the anisotropic conduction velocity in heart tissue. It is believed that anisotropic conduction is influenced by gap junction distribution and function, myocardial fibrosis, excitability, and cell size and shape [71]. Through modulation of PCL biofabrication and postprocessing techniques, anisotropy could be obtained in PCL scaffolds designed for heart tissues. Wanjare et al. reconfigured randomly oriented electrospun fibers using heat and uniaxial strain to create parallel electrospun PCL microfibers [72]. With the parallel-oriented fibers, better cardiomyocyte alignment and spontaneous beating were reported. Future research into fabricating PCL with precisely calibrated anisotropy and examining its effect on CMs will contribute to the understanding of the essential anisotropic characteristics needed in a designed cardiac patch.

### 3.2. Polyurethane-Based Patches (PU)

**Figure 5:**
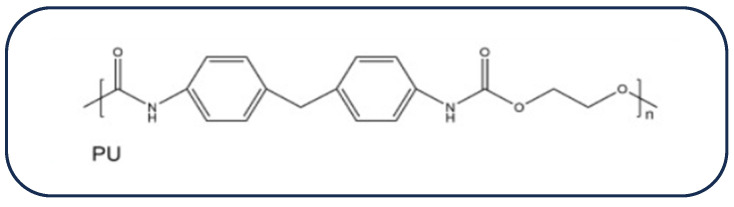
The chemical structure of PU.

Polyurethanes (PUs) represent a unique class of polymeric materials that differ significantly from many other plastic varieties. They can be found in a wide range of products, including paints, liquid coatings, integrated skins, elastomers, insulators, and elastic fibers [73]. Due to its exceptional mechanical characteristics, stability, and significant biocompatibility, PU is considered a favorable biomaterial for cardiac patch fabrication [23]. Figure 5 shows the chemical structure of the polymer polyurethane. Polyurethane exhibits a high degree of biocompatibility when implanted in the human body. As an example, Mani et al. fabricated a cardiac patch using a PU electrospun novel nanocomposite (PU/basil/TiO2) that was loaded with basil oil and titanium dioxide particles [74]. The produced patch demonstrated hemocompatibility by delaying coagulation times and providing a secure environment for red blood cells.

**Figure 6:**
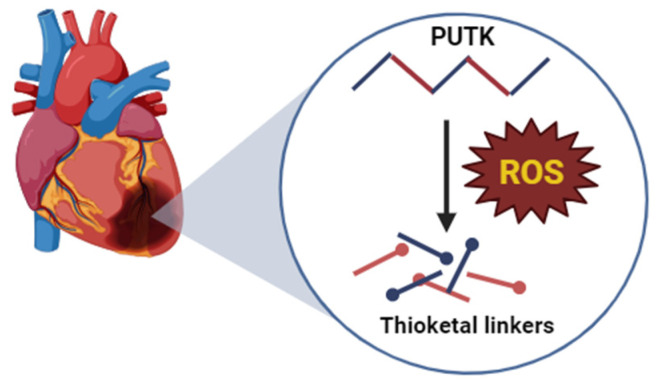
Schematic illustration of degradation of PUTK in response to ROS due to the cleavage of thioketal linkers.

The polymer chain of PU is primarily responsible for its considerable degradation time as well as other related physical and chemical characteristics. It is well established in the literature that urethane parts of PU do not emit any harmful degradation products. This is because a byproduct of the hydrolysis of urethane linkages is butanediamine [75]. Butanediamine, a polyamine found in all eukaryotic cells, is referred to as putrescine in the biomedical literature. Along with other polyamines, putrescine generally contributes to cell growth and proliferation [76]. In addition, the degradation byproducts of PU have been shown to play a vital role in improving cardiac function when implanted on the human heart [77]. Yao and colleagues developed a reactive oxygen species (ROS)-cleavable polyurethane patch (PU patch) using the ROS-cleavable thioketal linker (TK), which they subsequently loaded with methylprednisolone (MP) as an anti-inflammatory drug [78]. The elevated levels of ROS in the injured myocardium tissues caused the cleavage of the TK linker in the PU polymer. Consequently, there was a rapid degradation of the patch to release methylprednisolone, leading to a reduction in inflammation and a subsequent decrease in the infraction size, causing an overall improvement in cardiac function. Figure 6 demonstrates the degradation of PUTK in response to ROS due to the cleavage of thioketal linkers.

Poly (ester urethane)-urea (PEUU) scaffolds as PU-based materials have shown a greater improvement in cardiac function compared to other scaffolding designs. PEUU was implanted onto the infarcted left ventricle of Lewis rats by Wagner and coworkers [79]. The PEUU patch induced smooth muscle formation and caused cardiac muscle cellularization. As a result, there was a decrease in left ventricle adverse remodeling and an enhancement in the contractile function of damaged myocardial fibers [80]. In another study by Fujimoto et al. [81], PEUU scaffolds demonstrated host fibroblast penetration, endocardial endothelization, and reduced levels of inflammation after 4 weeks of implantation. In addition, the fact that PEUU was nearly entirely assimilated by the host at the 12-week mark suggests that the patch was successful in cultivating cells that could endure pressure without the need for extra structural support. Moreover, PEUU scaffolds have shown success in reducing the incidence of arrythmias when implanted onto a rat’s infarcted right ventricle wall. This held true when an angiogenic PEG fibrin-based hydrogel supplemented with an electrospun biodegradable PEUU mesh layer was examined in a study by Tao et al. [82]. The scaffold that was created promoted cell invasion, the growth of new blood vessels, and regenerative remodeling. With less fibrotic reaction and increased regenerative vascular and muscle remodeling, this designed PEUU-reinforced hydrogel patch improved heart function and reduced arrhythmia incidence. PEUU porous patches were implanted in pigs two weeks following induced MI in a study conducted by Fujimoto and co-workers [80]. The cardiac walls had thickened significantly after 8 weeks, showing increased vascularization and decreased development of scar tissue. A PU-based cardiac patch was shown to support cardiac gap junction formation in addition to demonstrating high elasticity and minimal deformation under mechanical tests [83].

Furthermore, it is hypothesized that the patch material’s porosity and hydrophobicity are crucial for the fusion and adhesion of cardiac cells to biomaterials. When compared with patches made of hydrophobic materials such as polytetrafluorethylene (ePTFE), PU-based patches exhibit superior hydrophilicity and improved mitochondrial activity after being loaded with cells [84]. This allows for better attachment and adhesion of cardiac cells to the PU scaffold. In addition, PU-based patches are capable of having abundant MSCs on their surface due to their high porosity, which resembles natural heart tissue [85]. Although the porous structure of PU-based patches allowed for the inclusion of more cells, the pore walls of the patches restricted intercellular communication and hindered the transmission of electrical signals [86]. Baheiraei and colleagues addressed this issue by incorporating conductive polyaniline into a polyurethane patch [84]. Since polyaniline serves as an electroactive substrate for electrically excitable cells, its incorporation resulted in electroactivity, with an electrical conductivity exceeding 10−5 S/cm, enhancing cell adhesion and proliferation [87]. Another important mechanical property of PU-based cardiac patches is anisotropy. Chen et al. [88] created a PU/cellulose composite scaffold for cardiac tissue engineering using electrospinning. The findings demonstrated the successful fabrication of anisotropic scaffolds with aligned nanofibers, which have expressive electroconductive ability necessary for cardiac cell survival and function.

### 3.3. Polyglycerol Sebacate (PGS)

**Figure 7:**
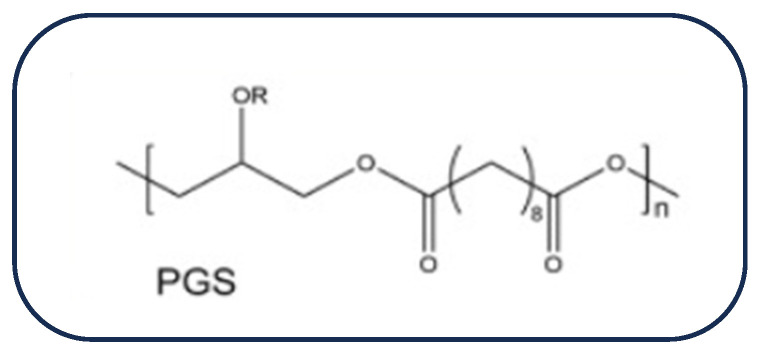
The chemical structure of PGS.

PGS is a cross-linked, amorphous polymer, which was created via the polycondensation reaction of glycerol and sebacic acid conducted by Wang et al. in 2002 [89]. Due to its numerous advantages, PGS has gained a lot of interest in the field of biomedical engineering. It has been commonly used in the fabrication of cardiac patches due to its affordability, significant biocompatibility, and exceptional mechanical characteristics that mimic myocardial fibers [90]. In addition, it has unique elastic properties which makes it favorable to endure the mechanical dynamics of the human heart [91]. Due to its unique chemical structure, cardiac patches made of PGS show a high degree of adhesion and attachment to the cardiac cells [92]. This is mainly because of the hydrophilicity of the polymer, which is attributed to the hydroxyl groups connected to the carbon backbone structurally [93]. The chemical structure of PGS is shown in Figure 7.

To create an optimal cardiac patch, the mechanical characteristics of PGS can be adjusted by modifying three processing parameters, which are the curing temperature, molar ratio of glycerol to sebacic acid, and curing time [36]. For example, through altering the curing time, the stiffness of the polymer could be changed. This was proven in a study done by Engelmayr, Jr. and co-workers, who fabricated a PGS-based cardiac patch with an accordion-like honeycomb structure [94]. They reduced the curing time to the point where the stiffnesses in the PGS patch’s distinct preferred (PD) direction and the cross-preferred (XD) direction were 83 ± 2 kPa and 31 ± 1 kPa, respectively. They were comparable to those of natural myocardial tissue, and consequently, the patch could adapt to the electrical and mechanical properties in different directions. With direction-dependent electrical stimulation, this anisotropic PGS patch could not only cause cardiomyocyte contraction, but it also had a better cell distribution than its isotropic equivalent [94]. In addition, PGS is an elastomeric material that has a high degree of flexibility, in which it can be fully recovered from substantial reversible deformation in mechanically dynamic circumstances [95]. Therefore, PGS is especially appealing for creating cardiac patches because of its flexibility. Marsano et al. [96] produced a porous PGS patch with a compressive modulus of 2.35 ± 0.03 kPa and a tensile modulus of 282 ± 25 kPa. This patch was sequentially perfused with cardiac myocytes, endothelial cells (ECs), and vascular endothelial growth factor (VEGF) by transduced myoblasts. This resulted in angiogenesis in the host myocardium as well as the patch, offering the possibility of creating a cardiac patch with vasculature [97].

Numerous studies have been conducted to understand PGS degradation in both in vitro and in vivo settings. It is well documented that the primary mechanism of degradation of PGS is surface degradation, in which cleavage of ester linkages takes place [98]. In PGS, which experiences surface degradation, there is a slow loss of mechanical strength (tensile properties) relative to mass loss (per unit original area). This is in contrast to bulk degradation mechanisms, for which the mechanical strength decreases in advance of mass loss, changing the geometry (shape and volume) of the polymer [99]. This slow loss of mechanical properties during degradation ensures sufficient time for the damaged cardiac cells to undergo a successful repairing process. The degradation of PGS-based patches has been studied in Sprague–Dawley rats with induced MI [100]. They found that the patch was totally absorbed with no granulation or scar tissue formation after being implanted subcutaneously. Additionally, the implantation site’s normal histological architecture was restored after 60 days following implantation [89]. Through altering different processing parameters such as the curing time and temperature, the degradation kinetics of PGS can be manipulated [93]. Chen and colleagues customized PGS degradation to correspond with the recovery kinetics of cardiac tissue [36]. While PGS synthesized at 130 °C showed no signs of degradation, PGS manufactured at 110 °C had faster degradation rates than PGS synthesized at 120 °C.

One of the main advantages of using PGS in the manufacture of cardiac patches is its considerable biocompatibility. Both in vivo and in vitro biocompatibility tests have shown that PGS is a promising candidate for various soft-tissue engineering applications [101]. Indeed, PGS’s biocompatibility is attributed to the fact that its initial reactants utilized in its manufacture are naturally biocompatible [102]. Sebacic acid is an essential metabolic intermediate in the ω-oxidation of medium- to long-chain fatty acids, while glycerol is the fundamental building block of lipids [103]. The body therefore spontaneously metabolizes the breakdown products of PGS. Furthermore, the PGS manufacturing process does not include the use of catalysts or additives, potentially preventing harmful consequences in biomedical applications [104]. Several studies have demonstrated the effect of biocompatible PGS cardiac patches in improving cardiac function. To stimulate cardiomyocyte proliferation, differentiation, and contractility in a nude rat model, Radisic et al. [105] constructed a PGS cardiac patch scaffold seeded with several cell types (Sprague–Dawley RCM and cardiac fibroblasts). Porogen leaching was used to generate these PGS scaffolds, which were then die-punched into disks with a 5 mm diameter and 2 mm thickness. The results showed that the PGS patch’s implantation induced cardiac fibroblast adhesion, which consequently promoted cardiomyocyte proliferation, differentiation, and contractility [106]. Additionally, PGS has even demonstrated success in bigger animal models; Ravichandran et al. [45] tested the effectiveness of a PGS scaffold as a biomimetic support of a porcine infarcted heart using a pig infarction model. Mesenchymal stem cells from bone marrow were used to precede the PGS and fibrinogen scaffolds that were placed in the left ventricle’s infarct bed. The materials used in this combination enhanced myocardial contractility and overall left-ventricular performance. Furthermore, the inclusion of cardiac marker proteins demonstrated that this scaffold was necessary for the proliferation and differentiation of bone-marrow-derived mesenchymal stem cells into cardiac cells [107].

### 3.4. Poly (L-Lactic Acid) (PLLA)

**Figure 8:**
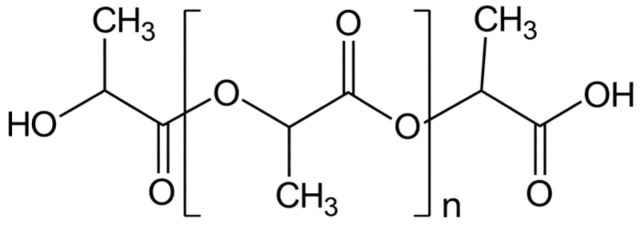
The chemical structure of PLLA.

Polylactic acid (PLA) is an aliphatic polyester that originates from lactic acid (2-hydroxypropionic acid) [108]. Lactic acid (2-hydroxy propionic acid), the fundamental component of PLA, can exist in optically active d- or l-enantiomers [109]. Therefore, PLA has two main stereoisomers, poly (L-lactide) acid (PLLA) and poly (D-lactide) acid (PDLA), or as a racemic mixture, designated as PDLLA. Based on the ratio of the enantiomers in the polymer, various material properties can be generated [110]. Besides adjusting the L/D ratio, there are numerous techniques to alter the characteristics of PLA, such as co-polymerizing it with other monomers (such caprolactone and glycolide), adding fillers and plasticizers, and applying heat treatments [111]. Due to its extraordinary mechanical and chemical characteristics, PLA has been widely use various biomedical applications, including soft-tissue implants, tissue engineering scaffolds and drug delivery devices [112,113]. The stereoisomer PLLA has proven to be highly biocompatible and biodegradable with exceptional mechanical strength and tremendous shaping and molding properties [114]. Therefore, it is commonly used as a scaffold for cardiac patch fabrication. The chemical structure of PLLA is illustrated in Figure 8.

One of PLLA’s most essential properties and the main driver of interest in its use in biomedical applications is its degrading behavior. The degradation time of PLLA is about 40 and 30 weeks in vitro and in vivo, respectively [115]. As a member of the polyester group, PLLA has a comparatively long hydrolysis half-life because of steric effects, in which the alkyl group prevents water from attacking its structure [116]. Therefore, PLLA scaffolds have a long degradation time, and hence, they provide adequate time for cardiac cells to undergo repair and regeneration. Another factor that influences the degradation rate of PLLA is the degree of crystallinity. This is because its crystalline groups are less permeable to water, and consequently, it undergoes slower hydrolysis [117]. PLLA fibers exhibit a high degree of crystallinity and a highly orientated microstructure. These properties cause the elongation to failure to vary between 20 and 100%, and the value of Young’s modulus normally ranges between 3 and 7 GPa [118]. In order to create a long-lasting polymeric patch, Ichihara et al. created a scaffold utilizing a sheet of PLLA with a copolymer of lactide acid and poly(lactide-co-caprolactone) (PLCL) [119]. The results showed that over a period of 6 months, the PLLA patch maintained its structural integrity in a high-pressure environment, with overall improvement in left-ventricular function and an increase in cardiac output.

Since PLLA has an extra methyl group, it remains structurally intact during degradation [120]. However, it comes at the expense of having a hydrophobic nature. Therefore, PLLA scaffolds have poor cellular adhesion and attachment due to their hydrophobicity. To increase PLLA’s hydrophilicity, electrospun PLLA scaffolds were created and modified with several ECM-derived proteins, including collagen, gelatin, fibronectin, and poly-L-lysine, as reported by Muniyandi et al. [121]. Significant cellular adhesion and proliferation of cardiac fibroblasts were observed on the modified PLLA scaffold, which supports the potential use of PLLA for cardiac patch fabrication. Furthermore, the addition of other polymers to PLA can improve its mechanical and chemical properties. For example, Flaig and colleagues developed an efficient electrospun scaffold for cardiac tissue engineering utilizing PLA and poly (glycerol sebacate) (PGS) elastomer [122]. Through adding PGS, the hydrophilicity of PLA increased, and there was notable improvement in surface functionalization. Furthermore, the morphology of the PLA: PGS scaffold containing cardiomyocytes resembled that of the original cardiac tissue. In general, the findings demonstrated the potential use of thin PLA: PGS scaffolds as biomaterials for cardiac patch fabrication.

One of the main advantages of PLLA is that it has a highly porous structure. The degree of porosity of PLLA ensures an extended release of drugs and growth factors necessary for the cardiac cell repair and healing process [123]. As an example, Chung et al. [124] fabricated an electrospun PLLA patch to develop an epicardial delivery system of cardiac stem cells (CSCs) and VEGF for the treatment of acute infarcted myocardium. The porous structure of the scaffold maintained a steady release of VEGF for four weeks, which enhanced the migration and proliferation of CSCs and endothelial cells. The results showed that there was a reduced amount of fibrosis in the infarcted area, which is attributed to the prolonged release of VEGF from the PLLA patch. Another study examined the use of a PLLA-based scaffold as a ventricular patch in a chronic myocardial infarction model in rabbits [125]. The scaffold was designed to release granulocyte-colony-stimulating factor (GCSF) to induce cellular proliferation, differentiation, and blood vessel formation. The post-implantation results showed that angiogenesis was successful in the functionalized PLLA scaffold, as evidenced by the scaffold’s ability to increase capillary density. Additionally, it showed a marked decrease in the incidence of ventricular dilation and improvement in cardiac performance.

## 4. Current Challenges and Future Perspectives

Optimal material selection serves as a foundational element for the eventual efficacy of cardiac patches. Although the polymers discussed in this review are under scrutiny by numerous research entities, their diverse properties significantly influence patch performance. These polymers exhibit distinct structures, leading to variable degradation rates. Each polymer can be customized in terms of structure and molecular weight to accommodate various degradation timeframes. All the polymers mentioned above (PCL, PGS, PU, and PLLA) exhibit excellent biocompatibility, rendering them highly desirable options for cardiac patches. Among these polymers, PGS is recognized for its ability to support extensive cell growth [125]. PU and PGS are also lauded for their exceptional physicochemical and mechanical properties, including heightened shear strength and elasticity [125]. In contrast, PLLA and PCL are relatively mechanically rigid [126]. Polyurethane (PU) fosters a cardiomyocyte-friendly environment due to its resistance to microbial and thrombotic factors [127]. Comparative studies have revealed that PCL exhibits superior cell retention and spreading compared to PLLA [128]. PGS exhibits outstanding biodegradability, ensuring complete post-implantation resorption [129]. Furthermore, the versatility in modifying these polymers enables convenient customization according to the unique demands of each patch.

Despite promising results in preclinical trials for cardiac repair, cardiac patches still face challenges prior to their clinical application [129]. While therapeutic integration benefits from their enhanced delivery efficiency, the majority of current cardiac patch implementations necessitate invasive open-chest surgery [130]. This poses a significant concern, especially for myocardial infarction patients who may struggle to recover from the surgical trauma and resulting inflammation, leading to heightened psychological distress [131]. Consequently, there is a critical need for less invasive delivery methods for these patches. Tang et al. have innovated a regenerative cardiac patch through the application of in situ polymerizable biomaterials via spray painting onto the heart’s surface [132]. However, there is a growing need for the investigation of even less invasive techniques to overcome this limitation. Additionally, improving implantation techniques and exploring innovative fabrication technologies and materials are essential steps forward. Advanced techniques like 3D printing and photoetching are recommended for patch fabrication, while materials featuring shape memory and superior mechanical properties hold promise as the next generation of biomaterials for cardiac patches [133].

A major challenge for clinical implementation is the effective integration of transplanted patches with the host myocardium. Several research groups have actively tackled this issue [134]. For instance, Tang and colleagues devised a polymeric microneedle patch combined with cardiac stem cells (CSCs) for therapeutic heart regeneration following acute myocardial infarction (MI) [135]. The microneedles acted as conduits, facilitating communication between the patch and the host myocardium. This enabled the transplanted patch to receive nutrients from the heart while releasing paracrine factors to aid in heart repair, ultimately promoting angiomyogenesis, reducing scar size, and enhancing cardiac function [136]. Their findings offer a promising approach to enhance patch integration with the host myocardium, and further research is anticipated to explore the specific characteristics of microneedles, including material composition, formulation techniques, and mechanical properties.

Another hurdle in the adoption of cardiac patch therapy is its biocompatibility. Although it has demonstrated success in animal models, the current biocompatibility of cardiac patches falls short of clinical requirements [137]. It is imperative to address the common occurrence of tissue adhesion following cardiac patch transplantation, leading to significant side effects. This issue stems from an immune response triggered by the foreign nature of the patches, posing a critical concern for clinical application. Researchers have identified that surface modification can significantly decrease tissue adhesion [138], making it a promising approach for polymer-based cardiac patches. Additionally, the biodegradation of the patches must be considered, as immune rejection persists unless the patches can degrade post-treatment [139]. Achieving such improvement necessitates materials that are inherently biodegradable without compromising therapeutic efficacy.

Furthermore, there is a crucial need for long-term storage to enable clinical application. Due to the intricate requirements of cell culture, current cell therapy utilizing cardiac patches is time-consuming and largely confined to laboratory settings [140]. Therefore, this technology demands strict adherence to industrial engineering ethics and standardization protocols to ensure the efficient and consistent production of these patches while also ensuring a reasonable shelf-life [141]. Overcoming the challenge of maintaining cell viability and functionality presents a significant obstacle to scaling-up the production of these therapeutic patches. Despite some studies indicating improvements in cell retention and engraftment with cardiac patches [142], their therapeutic efficacy remains below the clinical threshold. The quest for artificial materials with analogous functions to cells underscores the importance of delving deeper into the mechanistic aspects of cell therapy for heart regeneration.

In summary, an ideal synthetic cardiac patch for myocardial infarction should combine mechanical compliance that closely matches the native myocardium, controlled biodegradability synchronized with tissue remodeling, robust biocompatibility with minimal immune activation, and the capacity to support bioactive or cell-based therapeutic delivery. Polymer selection should therefore be guided by the primary therapeutic objective. For mechanical reinforcement of the infarcted ventricular wall, elastomeric polymers such as PGS and PU are more suitable due to their elasticity and shear strength, whereas relatively rigid polymers such as PCL and PLLA may provide structural support but require modification to better match myocardial mechanics. Conversely, for bioactive molecule or cell delivery, polymers with tunable degradation kinetics and favorable cell-interactive surfaces, such as PGS and modified PCL, may be more appropriate to enhance retention and paracrine signaling. Table S1 summarizes the key mechanical, degradation, and biological properties of major synthetic polymers commonly used in cardiac patch fabrication for myocardial infarction therapy.

## 5. Conclusions

Given the global prevalence of heart disease and its escalating incidence rates, regenerative therapies have become a primary focus of research aimed at improving outcomes for affected individuals. Despite the daily emergence of new opportunities driven by substantial research endeavors and funding allocations towards heart disease and cardiac patches, significant obstacles persist in the translation of research findings into clinical trials and eventual widespread therapeutic application. At present, the principal bottleneck preventing clinical translation is not solely material performance but the integration of the patch with host myocardium and the development of minimally invasive delivery strategies. Achieving electrical, mechanical, and biological coupling while avoiding open-chest surgery remains a central challenge. Therefore, future research should prioritize interface engineering, injectable or in situ forming systems, and scalable fabrication technologies that maintain reproducibility and regulatory compliance. A rational, goal-oriented polymer selection strategy combined with delivery innovation will be essential to advance synthetic cardiac patches from experimental platforms to clinically viable therapies. Currently, efforts are focused on combining different materials (scaffolds and embedded biological substances) and refining engineering fabrication methods to achieve optimal therapeutic outcomes with minimal side effects in animals with myocardial infarction (MI). However, with advancements in cell biology and engineering materials, there is optimism that cardiac patches may evolve into a comprehensive and efficacious treatment for heart diseases, thereby alleviating the burden and fatalities associated with cardiovascular diseases. In conclusion, the progress made in cardiac patches sets the stage for cardiac repair and serves as a source of inspiration for advancing heart regeneration.

## Data Availability

No new data were created or analyzed in this study.

## Supplementary Materials

The following supporting information can be downloaded at: https://www.mdpi.com/article/10.3390/biom16040580/s1, Table S1: Comparative Overview of Major Synthetic Polymers Used in Cardiac Patches.
