# Supplementary material for: Synthetic-Polymer-Based Cardiac Patches for MI-Induced Heart Failure Treatment: A Review
Source: Biomolecules. 2026 Apr 14;16(4):580. doi: 10.3390/biom16040580 (PMC13113715; doi:10.3390/biom16040580)
Supplement: Supplementary file 1 [file biomolecules-16-00580-s001.zip › biomolecules-4118544-supplementary.pdf]

Supplementary Table S1 summarizes the key mechanical, degradation, and biological properties of major synthetic polymers commonly used in cardiac patch fabrication for myocardial infarction therapy.

Polymer	Mechanical Profile	Degradation Rate	Biocompatibility	Key Advantages	Main Limitations	Best Suited For
**PCL**	Relatively rigid; moderate tensile strength	**Slow** – can persist ~2 years in vivo before significant molecular weight loss	Good	Excellent structural stability; good cell retention; easy processability	Long degradation time; limited elasticity vs. myocardium	Long-term mechanical reinforcement
**PU / PEUU**	Highly elastic; tunable modulus; strong shear resistance	**Moderate–Slow** – tunable depending on chemistry but generally slower than PLA/PLGA	Very good; thromboresistant	Excellent mechanical compliance; cardiomyocyte-friendly; customizable chemistry	Complex synthesis; long-term degradation products require evaluation	Mechanical support with dynamic compliance
**PGS**	Soft, elastomeric; myocardium-like elasticity	**Fast–intermediate** – can degrade fully in ~60 days in vivo (depending on crosslinking)	Excellent; supports cell proliferation	Biodegradable; favorable for cell growth; flexible	Lower mechanical strength compared to PU	Bioactive delivery; cell-based therapies
**PLLA**	Rigid; high tensile strength	**Intermediate–Slow** – months to ~1 year depending on crystallinity and in vivo conditions	Good	Structural integrity; well-established regulatory history	Brittleness; limited elasticity	Structural scaffolding requiring moderate degradation
